# Mitochondrial DNA Variation, Antiretroviral Therapy, and Incidence of Diabetes Among Men With and Without HIV

**DOI:** 10.1093/ofid/ofaf811

**Published:** 2026-01-03

**Authors:** Craig Cronin, Todd T Brown, Hsing-Yu Hsu, David C Samuels, Weiqun Tong, Sudipa Sarkar, Alison G Abraham, Jeremy J Martinson, Shehnaz K Hussain, Steven Wolinsky, Todd Hulgan, Jing Sun

**Affiliations:** Department of Medicine, Johns Hopkins University, Baltimore, Maryland, USA; Department of Epidemiology, Johns Hopkins University, Baltimore, Maryland, USA; Department of Medicine (Endocrinology), Johns Hopkins University, Baltimore, Maryland, USA; Department of Epidemiology, Johns Hopkins University, Baltimore, Maryland, USA; Department of Molecular Physiology and Biophysics, Vanderbilt School of Medicine, Nashville, Tennessee, USA; Department of Epidemiology, Johns Hopkins University, Baltimore, Maryland, USA; Department of Medicine (Endocrinology), Johns Hopkins University, Baltimore, Maryland, USA; Department of Epidemiology, Johns Hopkins University, Baltimore, Maryland, USA; Department of Infectious Diseases and Microbiology, Graduate School of Public Health, University of Pittsburgh, Pittsburgh, Pennsylvania, USA; Department of Public Health Sciences, School of Medicine and Comprehensive Cancer Center, University of California, Davis, California, USA; Department of Medicine (Infectious Diseases), Northwestern University, Chicago, Illinois, USA; Department of Medicine (Infectious Diseases), Vanderbilt University Medical Center, Nashville, Tennessee, USA; Department of Epidemiology, Johns Hopkins University, Baltimore, Maryland, USA

**Keywords:** aging, diabetes mellitus, HIV, mitochondrial genetics, mitochondrial-toxic antiretroviral therapy

## Abstract

**Background:**

Mitochondrial dysfunction is implicated in the development of diabetes mellitus (DM), which is more common in people with HIV (PWH) than in people without HIV (PWoH). Variation in mitochondrial DNA (mtDNA) and mitochondrial-toxic antiretroviral therapy (ART) may influence the susceptibility to DM but is underexplored in men with HIV.

**Methods:**

Men from the Multicenter AIDS Cohort Study (MACS) without DM and with fasting glucose data were included. Type 2 DM was defined by fasting glucose ≥ 126 mg/dL, DM medication use, a DM diagnosis, or hemoglobin A1c ≥ 6.5%. Exposure to mitochondrial-toxic ART (D-drugs or zidovudine) was categorized as a binary variable based on ever or never exposed. Mitochondrial DNA haplogroups were determined using HaploGrep from genotyping data. Associations between incident DM, mtDNA haplogroups of European and African origin, and interactions between mtDNA haplogroups and mitochondrial-toxic ART were analyzed.

**Results:**

Among 2598 men (667 self-reported as non-Hispanic Black and 1616 self-reported as non-Hispanic White), 1349 were men with HIV. In PWH, African haplogroup L3 was associated with a higher risk of incident DM (hazard ratio [HR], 1.92; 95% CI, 1.19–3.10) compared to other African-ancestry haplogroups, after adjusting for principal components of nuclear genetic ancestry, age, body mass index, hepatitis B and C status, smoking, and HIV-specific factors. D-drugs were independently associated with an increased risk of developing DM (HR, 2.8; 95% CI, 1.5–5.3).

**Conclusions:**

The African mtDNA haplogroup L3 increased the risk of incident DM in men with HIV. In PWH, D-drugs independently increased the risk of DM.

## Teaser

We assessed the role of mitochondrial DNA (mtDNA) variation and exposure to mitochondrial-toxic antiretroviral therapy in incident diabetes mellitus (DM) among men with and without HIV. African mtDNA haplogroup L3 and D-drug exposure were independently associated with increased DM risk in men with HIV.

As the life expectancy of people with HIV (PWH) approaches that of the general population, with greater than 50% of PWH now over 50 years old [1–5], many chronic diseases have become important challenges to address. Diabetes mellitus (DM) has a higher prevalence in PWH compared to people without HIV (PWoH) and results in 3-fold higher mortality, making it critical to study in this aging population [6, 7].

An important factor that has been implicated in DM pathophysiology is mitochondrial dysfunction [8, 9]. Mitochondria are known to be involved in impaired insulin release leading to DM [8], and a significant determinant of mitochondrial function is mitochondrial genetic variation. Mitochondrial DNA (mtDNA) haplogroups were originally developed through a comprehensive phylogenetic tree of global human mtDNA variation and can effectively trace ancestral lineages and geographic origins [10]. Mitochondrial DNA haplogroups have been associated with complex diseases, including various cancers, cardiovascular diseases, neurodegenerative diseases, and DM [11–17].

Independent of DM, HIV infection affects mitochondria through chronic inflammation [18], altered mitochondrial membrane potential [19, 20], and a reduced ability to repair DNA damage [19, 21], even in PWH who are ART naïve [19, 20]. We previously showed that African mtDNA haplogroup L2 was associated with lower risk of incident DM and haplogroup L3 was associated with faster decline in in β-cell function among women with HIV [9]. However, to our knowledge, the association of mtDNA haplogroups with risk of DM among men with HIV has not been published.

Another factor related to mitochondrial dysfunction in PWH is previous exposure to certain ART, namely nucleoside reverse transcriptase inhibitors (NRTIs) [22]. Of the NRTIs, D-drugs such as stavudine (D4T), zalcitabine (ddC), and didanosine (ddI) are known to have potent mitochondrial toxicity [23]. These drugs inhibit the mitochondrial-specific DNA polymerase (DNA pol-γ) impacting the ability of mitochondria to replicate [23, 24]. While mtDNA levels recover after changing to a less toxic therapy, mitochondrial dysfunction may persist for many years [24]. Literature on the interaction between mtDNA variations and prior D-drug exposure remains limited [25].

Our aim was to determine the relationship between mtDNA haplogroups and DM incidence in a cohort of men with, or at risk of, HIV. Additionally, we explored if there is an interaction between mtDNA haplogroups and D-drug or zidovudine (AZT) exposure and their association with incidence of DM.

## METHODS

### Study Population

The Multicenter AIDS Cohort Study (MACS) is a prospective cohort initiative that began in 1984 with a sample of males, with and without HIV, who have sex with males. The institutional review board at each of the 4 sites in the cohort (Baltimore/Washington, Chicago, Pittsburgh, and Los Angeles) approved the study protocol, and each participant provided informed written consent. In-depth descriptions of the study design, enrollment, and data collection are reported elsewhere [26, 27]. Briefly, there were 3 enrollment periods from 1984 to 2003 that recruited patients for semiannual visits with interviews, laboratory tests, and physical examinations. Fasting glucose (FG) and fasting insulin began to be routinely collected at semiannual visits as part of the MACS data collection protocols in 1999. The index visit was at the time of first FG and fasting insulin collection.

For this analysis, we included data from males who self-reported as non-Hispanic Black or non-Hispanic White. From this subpopulation, only participants who had genetic data available, had a FG plus A1c measurement at ≥2 visits, and did not have DM at the index visit were included.

### HIV Serostatus, HIV Disease Progression, and Antiretroviral Therapy Use

The diagnosis of HIV for men in this study was based on an initial enzyme-linked immunosorbent assay and secondarily confirmed by Western blot. Additional details on the serostatus and determination of disease progression in MACS has been previously discussed [11].

For PWH, exposure to current antiretroviral therapy (ART) regimen was self-reported at every study visit. We extracted prior exposure to mitochondrial-toxic ART, specifically AZT and D-drugs, which include stavudine, zalcitabine, and didanosine, from prospectively collected MACS data and summarized it as a binary variable based upon previous exposure. Use of first-generation protease inhibitors, including lopinavir, ritonavir, amprenavir, nelfinavir, saquinavir, and indinavir, was also recorded and summarized as a binary variable.

### Incidence of Type 2 Diabetes Mellitus

We included participants with FG and hemoglobin A1c data available for at least 2 visits. Participants who were diagnosed with DM at the index visit were excluded from the analysis. Incidence of type 2 DM was defined as either a FG ≥ 126 mg/dL, the use of DM medication, a DM diagnosis, or hemoglobin A1c ≥ 6.5%. Methods of glucose and hemoglobin A1c collection have been reported elsewhere [24, 28].

### Mitochondrial DNA Haplogroup Classification

Mitochondrial haplogroup determination methods have been reported previously [9, 11]. Briefly, mitochondrial DNA was analyzed for single nucleotide polymorphisms (SNPs) to group the participants into common haplogroups using HaploGrep between 2014 and 2015 [9, 11, 29]. Several genotyping platforms were utilized across MACS, including Illumina Human Hap 550, Illumina 1MDuo, Illumina 1M, and TaqMan. Although each platform includes a distinct set of mtDNA variants, haplogroups are defined by clusters of linked variants, enabling reliable assignment of major mitochondrial haplogroups. Using this approach, we identified common African (L1, L2, L3, and “other”) and European haplogroups or clades (H, UK, JT, IWX, or “other”) for analyses.

### Statistical Analyses

The data analysis was performed using SAS9.4. The characteristics of study participants at baseline were evaluated using the chi-square test for categorical variables and the Wilcoxon–Mann–Whitney test for continuous variables.

We used multivariate Cox regression to examine the association between common European (H, UK, JT, IWX, and “other”) or African haplogroups (L1, L2, L3, and “other”) and hazard of DM over time. Analyses were stratified by self-declared race. We further assessed the hazard of DM based on D-drug exposure. Within this analysis, we assessed if there was a statistical interaction between common haplogroups and D-drug exposure. The models were adjusted for principal components of nuclear genetic ancestry, age, body mass index (BMI), hepatitis B (HBV) and hepatitis C (HCV) status, and smoking. HIV-specific factors such as D-drug and AZT exposure, time-varying CD4+ cell counts, and the presence of detectable HIV RNA were additionally controlled in analyses in PWH. Covariate selections were based on review of literatures and preliminary findings of univariate analyses [9, 11, 30, 31]. Hepatitis B status was determined by the presence of HBV surface antigen, and HCV status was determined by detectable HCV RNA, both indicative of active infection.

A sensitivity analysis further adjusting for lipoatrophy measured closest to the index visit was conducted given prior MACS findings, demonstrating an association between certain mtDNA haplogroups and lipoatrophy [32]. Lipoatrophy was categorized as a binary variable (none or mild vs moderate or severe) based on the clinical subjective assessment of the face, arms, legs, and buttocks, with each individual classified according to the most severe grade across these regions. Lipohypertrophy prevalence was similarly defined as a binary variable using the same body sites and subjective clinical assessment.

## RESULTS

### Characteristics of Participants at Enrollment

**Figure 1.**
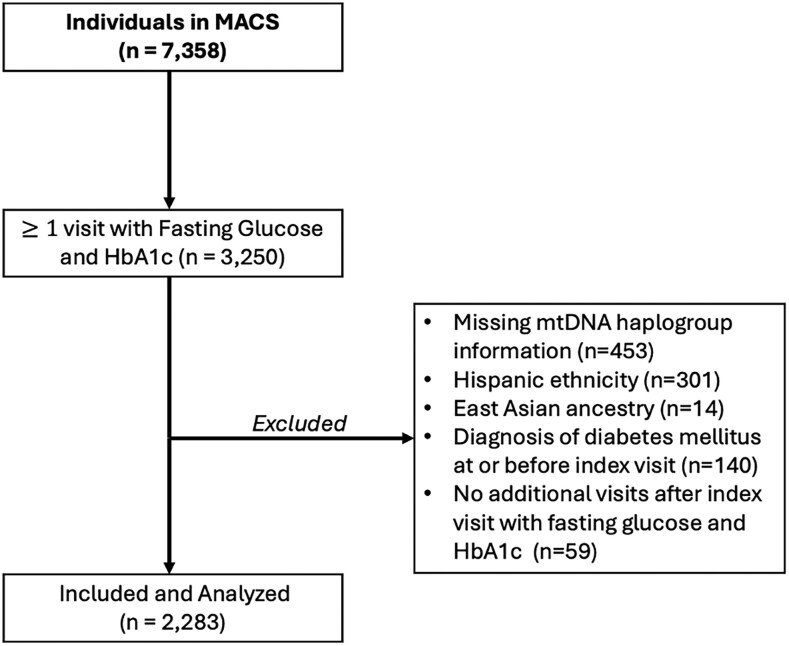
Flowchart of inclusion and exclusion criteria for the analytic sample from the MACS cohort.

Of the 2283 men included in the study shown in Figure 1, 667 self-reported as non-Hispanic Black with a median follow-up of 8.54 years (interquartile range [IQR], 4.0–15.8), and 1616 self-reported as non-Hispanic White with a median follow-up of 14.89 years (IQR, 6.3–17.5). The median age at the index visit was 45 years (IQR, 39–51) with a median BMI of 26 (IQR, 23–28). At the index visit, 11.3% of the cohort had lipoatrophy. Among all participants, 1145 (50%) were men living with HIV, including 424 who self-reported as non-Hispanic Black and 721 who self-reported as non-Hispanic White. Among PWH, 335 (29.3%) men were being treated with D-drugs and 319 (27.9%) were taking AZT at their index visits. The prevalence of lipoatrophy at the index visit in PWH was 20.1%, while only 1.8% in PWoH. Additional demographic and baseline characteristics are detailed in Table 1, with stratification by self-reported race in Supplementary Tables 1 and 2.

**Table 1.** Characteristics of Study Participants at Enrollment and Mitochondrial DNA Haplogroup Frequencies

All	Overall (n = 2283)	HIV− (n = 1138)	HIV+ (n = 1145)
Age at index visit, years	45 (39, 51)	47 (40, 53)	43 (37, 49)
BMI, kg/m^2^	26 (23, 28)	26 (24, 29)	25 (23, 28)
CD4+ count, cells/mm^3^	723 (491, 970)	901 (716, 1093)	515 (343, 735)
HIV RNA, copies/mL	…	…	50 (50, 6193)
Viral load	…	…	…
Detectable	…	…	446 (39.0%)
Undetectable	…	…	691 (60.3%)
Missing	…	…	8 (0.7%)
On ART at index visit	…	…	…
Yes	…	…	818 (71.4%)
No	…	…	316 (27.6%)
Missing	…	…	11 (1.0%)
Total cholesterol, mg/dL	190 (162, 219)	193 (167, 221)	188 (158, 218)
LDL cholesterol, mg/dL	113 (90, 139)	118 (95, 143)	109 (85, 134)
HDL cholesterol, mg/dL	46 (39, 55)	49 (41, 58)	43 (36, 52)
Triglycerides, mg/dL	126 (83, 195)	108 (75, 163)	150 (100, 254)
Glucose, mg/dL	90 (83, 97)	90 (82, 97)	90 (83, 98)
Lipoatrophy at index visit	…	…	…
Yes	258 (11.3%)	20 (1.8%)	238 (20.1%)
No	1723 (75.5%)	1062 (93.3%)	661 (55.7%)
Missing	302 (13.2%)	56 (4.9%)	288 (24.3%)
Lipohypertrophy at index visit	…	…	…
Yes	393 (17.2%)	180 (17.8%)	213 (16.8%)
No	1604 (70.3%)	778 (76.9%)	826 (65.0%)
Missing	286 (12.5%)	54 (5.3%)	232 (18.3%)
Index visit year	2002 (2000, 2003)	2002 (2001, 2003)	2002 (2000, 2005)
Most recent visit year	2018 (2010, 2019)	2018 (2010, 2019)	2018 (2010, 2019)
**Non-Hispanic Black**	Overall (n = 667)	HIV− (n = 243)	HIV+ (n = 424)
Median follow-up time: 8.54 y			
African mtDNA haplogroups	…	…	…
L1, n (%)	128 (19.2)	40 (17.9)	88 (19.9)
L2, n (%)	214 (32.1)	64 (28.6)	150 (33.9)
L3, n (%)	253 (37.9)	89 (39.7)	164 (37.0)
Other, n (%)	72 (10.8)	31 (13.8)	41 (9.3)
**Non-Hispanic White**	Overall (n = 1616)	HIV− (n = 895)	HIV+ (n = 721)
Median follow-up time: 14.89 y			
European mtDNA haplogroups	…	…	…
UK, n (%)	400 (24.8)	237 (27.7)	163 (21.5)
JT, n (%)	326 (20.2)	168 (19.6)	158 (20.8)
H, n (%)	725 (44.9)	361 (42.1)	364 (48.0)
IWX, n (%)	98 (6.1)	56 (6.5)	42 (5.5)
Others, n (%)	67 (4.1)	35 (4.1)	32 (4.2)

Values shown are median (IQR) or n (%), unless otherwise indicated.

Abbreviations: ART, antiretroviral therapy; BMI, body mass index; HDL, high-density lipoprotein; IQR, interquartile range; LDL, low-density lipoprotein; mtDNA, mitochondrial DNA.

Overall, men living with HIV were younger and had lower total cholesterol and high-density lipoprotein (HDL) levels compared to men living without HIV. Among non-Hispanic Black men, the most common mitochondrial haplogroups were L3 (37.9%) and L2 (32.1%). For non-Hispanic White men, the H (44.9%) and UK (24.8%) haplogroups were predominant (Table 1).

### Associations Between African Mitochondrial DNA Haplogroups and Incident Diabetes Mellitus

Using a univariate Cox proportional hazards model, we observed that the African haplogroup L3 was associated with a significantly higher risk of developing DM compared to other haplogroups among non-Hispanic Black men living with HIV. In the adjusted model, PWH with the L3 haplogroup had a 92% higher hazard (hazard ratio [HR], 1.92; 95% CI, 1.19–3.10) of developing DM during follow-up compared to those with non-L3 haplogroups (Table 2). In PWoH, no significant differences in DM incidence were observed across mitochondrial haplogroups.

**Table 2.** Unadjusted and Adjusted Longitudinal Associations Between Specific African or European Haplogroups and Incident Diabetes Mellitus

	All				HIV−				HIV+			
	Univariate		Multivariate^a^		Univariate		Multivariate^a^		Univariate		Multivariate^b^	
Haplogroup	HR (95% CI)	*P* value	HR (95% CI)	*P* value	HR (95% CI)	*P* value	HR (95% CI)	*P* value	HR (95% CI)	*P* value	HR (95% CI)	*P* value
African L2 versus non L2	0.78 (0.55, 1.16)	0.24	0.81 (0.54, 1.21)	0.30	1.08 (0.56, 1.81)	0.98	1.09 (0.58, 2.06)	0.78	0.70 (0.43, 1.14)	0.15	0.67 (0.38, 1.17)	0.16
African L3 versus non L3	1.52 (1.09, 2.13)	0.01	1.55 (1.08, 2.22)	0.02	1.00 (0.58, 1.71)	0.99	1.04 (0.57, 1.88)	0.90	1.96 (1.28, 3.02)	0.002	1.92 (1.19, 3.10)	0.007
European UK versus non-UK	1.26 (0.96, 1.65)	0.10	1.37 (1.02, 1.84)	0.04	1.23 (0.85, 1.78)	0.28	1.41 (0.94, 2.14)	0.10	1.35 (0.91, 2.00)	0.13	1.37 (0.88, 2.13)	0.17

Abbreviations: −, seronegative; +, seropositive; HR, hazard ratio.

^a^Multivariate models adjusted for age, BMI, HCV or HBV infection, smoking status, and principal components of nuclear genetic ancestry.

^b^Multivariate models adjusted for age, BMI, HCV or HBV infection, smoking status, principal components of genetic ancestry, AZT exposure, D-drug exposure, CD4 count, and detectable HIV RNA.

In a sensitivity analysis including the presence of moderate or severe lipoatrophy at the index visit to the adjusted model, PWH with the L3 haplogroup had a 101% higher hazard (HR, 2.02; 95% CI, 1.24–3.27) of developing DM compared to those with non-L3 haplogroups (Supplementary Table 3). When further restricting to individuals with no previous use of first-generation protease inhibitors, which are known to cause lipoatrophy, the results did not significantly change (Supplementary Table 4).

### Associations Between European Mitochondrial DNA Haplogroups and Incident Diabetes Mellitus

In a similarly adjusted Cox proportional hazards model including all non-Hispanic Whites, men with the UK haplogroup had a significantly higher risk of developing DM compared to those with non-UK haplogroups (HR, 1.37; 95% CI, 1.02–1.84; Table 2). After stratifying by HIV status, no significant difference in DM risk was observed for men with the European UK haplogroup (Table 2). No significant differences in DM incidence were observed among men with other European haplogroups (JT, H, IWX, and “other”).

In 2 sensitivity analyses including presence of moderate or severe lipoatrophy at the index visit to the adjusted model as well as restricting to individuals without prior use of first-generation protease inhibitors, the results for PWH and the UK haplogroup did not significantly change (Supplementary Tables 3 and 4).

### Association Between D-Drug and Zidovudine Exposure and Incident Diabetes Mellitus

**Figure 2.**
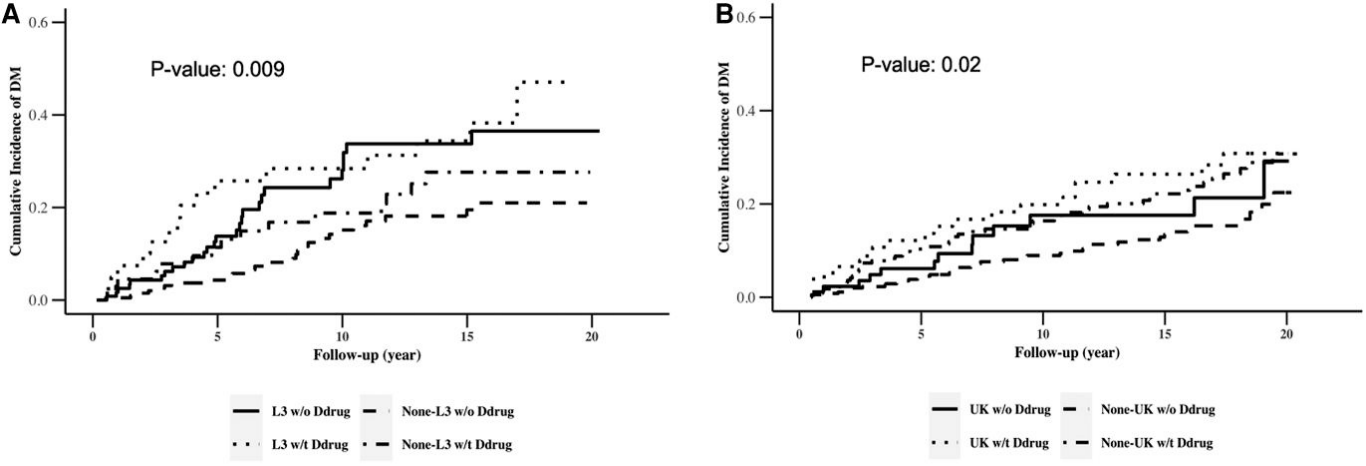
Cumulative incidence of DM by mitochondrial DNA haplogroups and exposure to D-drug antiretroviral therapy in the MACS. *A*, Cumulative incidence of DM by mitochondrial DNA haplogroup L3 and exposure to D-drug antiretroviral therapy among MACS participants living with HIV. *B*, Cumulative incidence of DM by mitochondrial DNA haplogroup UK and exposure to D-drug antiretroviral therapy among MACS participants living with HIV.

No significant interactions were observed between D-drug exposure and mitochondrial haplogroups in relation to incident DM. However, PWH with previous D-drug exposure had a significantly higher independent risk of developing DM during the follow-up period (HR, 2.8; 95% CI, 1.5–5.3). Table 3 summarizes the risk of developing DM by D-drug exposure and mtDNA haplogroups. Among non-Hispanic Black men, those with the haplogroup L3 and prior D-drug exposure developed DM the fastest (median 3.5 years; Figure 2*A*), whereas men with non-L3 haplogroups and without D-drug exposure had the longest time to DM development (median 7 years; Figure 2*A*). The independent effect of AZT use and first-generation protease inhibitors on incident DM was not statistically significant, and no interactions between AZT exposure or first-generation protease inhibitors and mitochondrial haplogroups were detected.

**Table 3.** Unadjusted and Adjusted Hazard Ratios for Incident Diabetes Mellitus Among HIV-Positive Individuals Grouped by D-Drug Exposure and Specific Mitochondrial Haplogroups

Group	Comparison	Univariate	Multivariate^a^
		HR (95% Wald Confidence Limits)	HR (95% Wald Confidence Limits)
D-drug (−)	African L3 versus non-L3	1.94 (1.21, 3.10)	1.98 (1.17, 3.35)
D-drug (−)	European UK versus non-UK	1.36 (0.87, 2.11)	1.38 (0.84, 2.28)
D-drug (+)	African L3 versus non-L3	2.00 (0.67, 5.97)	1.71 (0.56, 5.20)
D-drug (+)	European UK versus non-UK	1.34 (0.57, 3.18)	1.32 (0.52, 3.36)
African non-L3	D-drug (+) versus D-drug (−)	2.25 (0.84, 5.43)	3.02 (1.20, 7.63)
African L3	D-drug (+) versus D-drug (−)	2.33 (1.02, 5.28)	2.61 (1.11, 6.15)
European non-UK	D-drug (+) versus D-drug (−)	2.09 (1.23, 3.55)	2.04 (1.13, 3.68)
European UK	D-drug (+) versus D-drug (−)	2.07 (0.88, 4.87)	1.95 (0.75, 5.06)

Abbreviations: −, no previous D-drug exposure; +, previous D-drug exposure; HR, hazard ratio.

^a^Multivariate models controlled for age, BMI, HCV or HBV infection, smoking status, principal components of genetic ancestry, AZT, D-drug, CD4 count, and detectable HIV RNA.

## DISCUSSION

Using data from a large, prospective cohort study of men from European or African ancestry, we observed that the African haplogroup L3 was associated with a significantly higher risk of developing DM among PWH compared to those with non-L3 haplogroups. Additionally, exposure to mitochondrial toxic ART, specifically D-drugs, was independently associated with an increased risk of developing DM. Although a statistically significant interaction between D-drugs and haplogroups was not observed, their combined effects on DM risk were aggregative. People with HIV with haplogroup L3 and prior D-drug exposure developed DM in the shortest time. These results suggest that certain mtDNA haplogroups and D-drug exposure may independently contribute to an increased risk of DM in PWH, likely through their effects on mitochondrial function.

Our previous work from the Women's Interagency HIV Study (WIHS) found similar results among women with HIV, where the L3 haplogroup was associated with a significantly higher risk of developing DM compared to non-L3 haplogroups [9]. In contrast, women with HIV and the L2 haplogroup had the lowest risk of developing DM, accompanied by significantly slower β-cell function decline compared to non-L2 haplogroups [9]. These findings suggest a potential protective role of the L2 haplogroup in women living with HIV. Although the L2 haplogroup showed a trend toward a protective effect in men living with HIV in our study, the association was not statistically significant. This discrepancy may be explained by differences in sample size: WIHS included 269 PWH with the L2 haplogroup, compared to 150 PWH with the L2 haplogroup in our study. Nevertheless, the L3 haplogroup appears to increase DM risk across sexes in PWH, while L2 may confer some protective benefits. Future studies in other diverse populations are needed to validate these findings and investigate potential sex-specific interactions with haplogroups in relation to DM risk.

Among European haplogroups, only haplogroup UK was significantly associated with an altered risk of DM in non-Hispanic White men, though prior research has yielded varied findings regarding other European mtDNA haplogroups and metabolic dysfunction in PWH and PWoH [32–35]. For example, a study in a Brazilian cohort of the general population reported an association between haplogroup clade JT and DM, while another study using the MACS cohort found that men living with HIV and haplogroup J had a more rapid decline in gait speed compared to other European haplogroups [11, 15].

Our findings that men with haplogroup UK had a higher risk of developing DM may be supported by a cellular study showing that this haplogroup exhibited lower mtDNA levels, reduced mitochondrial protein synthesis, fewer electron transport chain units, and decreased mitochondrial membrane potential under stress compared to haplogroup H [36]. Among PWH coinfected with HCV, haplogroup UK has also been associated with insulin resistance [30]. Although we did not observe a statistically significant association between haplogroup UK and DM risk in PWH, the HR was relatively large (HR, 1.37). The lack of statistical significance in our stratified model may be due to limited power resulting from the reduced sample size. Further research into mitochondrial genetic variations associated with haplogroup UK, particularly in the context of HIV-related mitochondrial dysfunction [18–21], could provide valuable insights into its potential role in metabolic dysfunction and DM development.

Among PWoH, no significant differences in the risk of developing DM were observed across mitochondrial haplogroups, consistent with our previous findings from the WIHS study with women living with and without HIV [9]. However, some studies in the general population have identified associations between certain haplogroups and metabolic disorders such as DM [33–36]. The heterogenous association between haplogroups and DM risk observed in our study could suggest that HIV or ART exposure may serve as a major effect modifier in this relationship. It remains unclear whether this finding reflects reduced mitochondrial stress in PWoH, making genetic differences in mitochondria less pronounced, or whether an interaction exists between HIV-related mitochondrial effects and underlying mitochondrial genetic differences. Understanding this interplay is critical for future research, as it may help elucidate the mechanisms driving these associations and inform clinical targets, treatment strategies, and monitoring guidelines, particularly for high-risk haplogroups prone to DM or other metabolic disorders.

Mitochondrial dysfunction, insulin resistance, and an increased risk of developing DM associated with NRTIs, specifically D-drugs, are well described [22–24, 37–39]. Our findings reinforce that D-drug exposure independently increases the risk of developing DM, and these legacy effects may persist years after exposure to D-drugs. While D-drugs exert the most pronounced mitochondrial effects, newer ART have also been shown to influence mitochondrial function [40, 41]. Additional studies incorporating direct measurements of mitochondrial function and insulin resistance with additional types of ART could clarify the mechanisms through which these therapies, HIV, and mtDNA haplogroups contribute to metabolic dysfunction.

Our study has several limitations. First, while our study has a robust sample size overall, each individual haplogroup in European and African ancestry is further reduced, limiting the power to detect potential interactions between D-drug exposure and mitochondrial haplogroup on incident DM. Second, the study did not explore subhaplogroups, which may provide a more nuanced understanding of mitochondrial genetic influences on DM risk. Third, although the MACS cohort maintains a visit-by-visit retention rate of over 90%, loss to follow-up could introduce bias by disproportionately excluding sicker individuals who might experience additional metabolic complications, possibly underestimating the incidence of DM. Additionally, generalizability may be limited as the cohort consisted of men from urban MACS sites, with a median baseline age of 44 years. Lastly, other risk factors that may influence DM risk among PWH, such as chronic inflammation, certain coinfections, nuclear genetic variation, use of medications such as statins, and nutritional factors, could represent potential unmeasured confounders in this study. Despite these limitations, this study is a large, prospective cohort that enabled us to conduct the first longitudinal study evaluating incident DM in relation to mitochondrial haplogroups for men with and without HIV of European and African ancestry. Moreover, the similar sociodemographic and behavior characteristics between men with and without HIV minimized confounding effects.

In summary, non-Hispanic Black men living with HIV of African ancestry with the mitochondrial haplogroup L3 had an increased risk of developing DM. Prior D-drug exposure was independently associated with an elevated risk of developing DM in PWH. The combined effects of mitochondrial dysfunction from HIV and D-drug exposure appear to place haplogroup L3 individuals at the greatest risk of developing DM. Further research is needed to elucidate the independent and interactive roles of D-drugs, HIV, and mitochondrial haplogroups in metabolic dysfunction. Such studies may identify novel therapeutic targets and prevention strategies to mitigate the burden of DM and related metabolic comorbidities in people living with HIV.

## Supplementary Material

ofaf811_Supplementary_Data

## Supplementary Data

Supplementary materials are available at *Open Forum Infectious Diseases* online. Consisting of data provided by the authors to benefit the reader, the posted materials are not copyedited and are the sole responsibility of the authors, so questions or comments should be addressed to the corresponding author.
