# Supplementary material for: Mitochondrial DNA Variation, Antiretroviral Therapy, and Incidence of Diabetes Among Men With and Without HIV
Source: Open Forum Infect Dis. 2026 Jan 3;13(1):ofaf811. doi: 10.1093/ofid/ofaf811 (PMC12817993; doi:10.1093/ofid/ofaf811)
Supplement: ofaf811_Supplementary_Data [file ofaf811_supplementary_data.docx]

**Supplementary Table 1.** Characteristics of Study Participants Self-Reported as non-Hispanic Black at Enrollment

|  | Overall  (n=667) | HIV-  (n=243) | HIV+  (n=424) | P-value |
| --- | --- | --- | --- | --- |
| Age at visit, median (IQR) | 40(34, 46) | 41(35, 46) | 40(33, 46) | 0.2835 |
| BMI, median (IQR) | 25(23, 29) | 26(24, 29) | 25(23, 28) | 0.0013 |
| CD4, median (IQR) | 648(425, 914) | 903(718, 1119) | 497(340, 735) | <.0001 |
| HIV RNA, median (IQR) | 171(50, 11024) | - | 171(50, 11024) | - |
| Viral load |  |  |  |  |
| Detectable | - | - | 197 (46.5%) | - |
| Undetectable | - | - | 222 (52.4%) | - |
| Missing | - | - | 5 (1.2%) | - |
| ART |  |  |  |  |
| Yes | - | - | 158 (37.3%) | - |
| No | - | - | 265 (62.5%) | - |
| Missing | - | - | 1 (0.2%) | - |
| Total Cholesterol, median (IQR) | 172(150, 205) | 178(155, 213) | 169(147, 201) | 0.0021 |
| LDL direct, median (IQR) | 93(80, 113) | 88(88, 132) | 94(79, 111) | 0.8651 |
| HDL, median (IQR) | 48(40, 58) | 51(43, 60) | 46(39, 56) | <.0001 |
| Triglyceride, median (IQR) | 88(81, 96) | 89(82, 96) | 88(81, 96) | 0.3193 |
| Glucose, median (IQR) | 11(8, 17) | 12(8, 16) | 11(8, 17) | 0.8786 |
| Lipoatrophy at index visit |  |  |  |  |
| Yes | 41 (6.1%) | 7 (2.9%) | 34 (8.0%) |  |
| No | 499 (74.8%) | 222 (91.4%) | 277 (65.3%) |  |
| Missing | 127 (19.0%) | 14 (5.8%) | 113 (26.7%) |  |
| Lipohypertrophy at index visit |  |  |  |  |
| Yes | 103 (15.4%) | 36 (30.8%) | 67 (31.9%) |  |
| No | 443 (66.4%) | 67 (57.3%) | 36 (17.1%) |  |
| Missing | 121 (18.1%) | 14 (12.0%) | 107 (51.0%) |  |

**Supplementary Table 2.** Characteristics of Study Participants Self-Reported as non-Hispanic White at Enrollment

|  | Overall  (n=1616) | HIV-  (n=895) | HIV+  (n=721) | P-value |
| --- | --- | --- | --- | --- |
| Age at visit, median (IQR) | 47(41, 52) | 48(42, 54) | 45(39, 50) | <.0001 |
| BMI, median (IQR) | 26(23, 28) | 26(24, 29) | 25(23, 27) | <.0001 |
| CD4, median (IQR) | 746(524, 990) | 898(715, 1087) | 522(349, 737) | <.0001 |
| HIV RNA, median (IQR) | - | - | 50(50, 3428) | - |
| Viral load |  |  |  |  |
| Detectable | - | - | 249 (34.5%) | - |
| Undetectable | - | - | 469 (65.0%) | - |
| Missing | - | - | 3 (0.4%) | - |
| ART |  |  |  |  |
| Yes | - | - | 158 (21.9%) | - |
| No | - | - | 553 (76.7%) | - |
| Missing | - | - | 10 (1.4%) | - |
| Total Cholesterol, median (IQR) | 197(170, 225) | 196(171, 224) | 198(168, 226) | 0.9217 |
| LDL direct, median (IQR) | 100(80, 126) | 92(80, 128) | 101(80, 126) | 0.9855 |
| HDL, median (IQR) | 46(38, 54) | 49(41, 57) | 42(35, 51) | <.0001 |
| Triglyceride, median (IQR) | 90(83, 98) | 90(82, 97) | 91(84, 99) | 0.0003 |
| Glucose, median (IQR) | 11(8, 16) | 10(8, 14) | 12(9, 18) | <.0001 |
| Lipoatrophy at index visit |  |  |  |  |
| Yes | 217 (13.4%) | 13 (1.5%) | 204 (26.7%) |  |
| No | 1224 (75.7%) | 840 (93.9%) | 384 (50.3%) |  |
| Missing | 175 (10.8%) | 42 (4.7%) | 175 (22.9%) |  |
| Lipohypertrophy at index visit |  |  |  |  |
| Yes | 290 (17.9%) | 144 (16.1%) | 146 (20.2%) |  |
| No | 1161 (71.8%) | 711 (79.4%) | 450 (62.4%) |  |
| Missing | 165 (10.2%) | 40 (4.5%) | 125 (17.3%) |  |

**Supplementary Table 3.** Sensitivity Analysis of Adjusted Model Including Moderate or Severe Lipoatrophy at Index Visit for Associations between Specific African or European Haplogroups and Incidence Diabetes Mellitus among Men with HIV

|  | Multivariate^a^ | |
| --- | --- | --- |
| Haplogroup | HR  (95% CI) | *P*value |
| African L2 vs. non L2 | 0.65 (0.37, 1.15) | 0.14 |
| African L3 vs. non L3 | 2.02 (1.24, 3.27) | 0.005 |
| European UK vs. non-UK | 1.25 (0.78, 2.01) | 0.35 |

Abbreviations: CI, confidence interval; HIV, human immunodeficiency virus; HR, hazard ratio.

^a^Multivariate Models adjusted for age, BMI, HCV or HBV infection, smoking status, principal components of genetic ancestry, AZT exposure, D-drug exposure, CD4 count, detectable HIV RNA, and lipoatrophy (yes/no) at index visit

**Supplementary Table 4.** Sensitivity Analysis of Adjusted Model Including Moderate or Severe Lipoatrophy at Index Visit Restricted to Individuals without Prior use of Protease Inhibitors for Associations between Specific African or European Haplogroups and Incidence Diabetes Mellitus among Men with HIV

|  | Multivariate^a^ | |
| --- | --- | --- |
| Haplogroup | HR  (95% CI) | *P*value |
| African L2 vs. non L2 | 0.32 (0.093, 1.09) | 0.069 |
| African L3 vs. non L3 | 3.93 (1.73, 8.95) | 0.001 |
| European UK vs. non-UK | 1.92 (0.73, 5.06) | 0.19 |

Abbreviations: CI, confidence interval; HIV, human immunodeficiency virus; HR, hazard ratio.

^a^Multivariate Models adjusted for age, BMI, HCV or HBV infection, smoking status, principal components of genetic ancestry, AZT exposure, D-drug exposure, CD4 count, detectable HIV RNA, and lipoatrophy (yes/no) at index visit
